# Global Metabolic Responses to Salt Stress in Fifteen Species

**DOI:** 10.1371/journal.pone.0148888

**Published:** 2016-02-05

**Authors:** Daniel C. Sévin, Jacqueline N. Stählin, Georg R. Pollak, Andreas Kuehne, Uwe Sauer

**Affiliations:** 1 Institute of Molecular Systems Biology, ETH Zürich, Zurich, Switzerland; 2 PhD Program on Systems Biology, Life Science Zurich, Zurich, Switzerland; Hainan University, CHINA

## Abstract

Cells constantly adapt to unpredictably changing extracellular solute concentrations. A cornerstone of the cellular osmotic stress response is the metabolic supply of energy and building blocks to mount appropriate defenses. Yet, the extent to which osmotic stress impinges on the metabolic network remains largely unknown. Moreover, it is mostly unclear which, if any, of the metabolic responses to osmotic stress are conserved among diverse organisms or confined to particular groups of species. Here we investigate the global metabolic responses of twelve bacteria, two yeasts and two human cell lines exposed to sustained hyperosmotic salt stress by measuring semiquantitative levels of hundreds of cellular metabolites using nontargeted metabolomics. Beyond the accumulation of osmoprotectants, we observed significant changes of numerous metabolites in all species. Global metabolic responses were predominantly species-specific, yet individual metabolites were characteristically affected depending on species’ taxonomy, natural habitat, envelope structure or salt tolerance. Exploiting the breadth of our dataset, the correlation of individual metabolite response magnitudes across all species implicated lower glycolysis, tricarboxylic acid cycle, branched-chain amino acid metabolism and heme biosynthesis to be generally important for salt tolerance. Thus, our findings place the global metabolic salt stress response into a phylogenetic context and provide insights into the cellular phenotype associated with salt tolerance.

## Introduction

Preventing lysis and maintaining intracellular solute concentration homeostasis in the face of unpredictably changing environments are major challenges to all cells. Since cytoplasmic membranes are permeable to water molecules but restrict the diffusion of larger and polar compounds, changes in extracellular concentrations of non-permeating solutes cause changes in the hydrostatic (turgor) pressure difference between cytoplasm and surrounding medium [1]. When extracellular solutes suddenly deplete, water molecules enter the cell and thereby increase its turgor pressure. The resulting mechanical strain ultimately ruptures the cell envelope, leading to cell lysis. Conversely, accumulating extracellular solutes cause water molecules to exit the cell, thereby reducing its turgor pressure and intracellular water activity. This alters thermodynamic properties of the cytoplasm, causes protein misfolding and leads to macromolecular crowding, thereby affecting the rates of various cellular processes ranging from protein-DNA binding to metabolic reactions [2–6]. Maintaining turgor pressure and water activity in a physiologically tolerable range is therefore crucial for cells to survive and thrive in habitats with changing osmolalities.

Various evolved strategies enable cells to cope with osmotic stress. Most microorganisms surround their fragile cytoplasmic membrane with a rigid cell wall to increase the range of tolerable turgor pressures upon osmotic downshifts [7]. The increase of turgor pressure itself is limited by constitutively expressed mechanosensitive channels in the cytoplasmic membrane that release intracellular solutes nonspecifically when membrane strain exceeds critical values [8]. Furthermore, osmotic stress can cause alterations of cytoplasmic membrane properties such as permeability or stability, for example through cardiolipin or isoprenoid accumulation [9,10]. Moreover, cells are able to actively adjust the osmolality of their cytoplasm by regulating their intracellular solute concentration. Following an osmotic upshift, inorganic ions such as K^+^ and organic counter-ions such as glutamate rapidly accumulate in the cytoplasm [11,12]. Once inorganic ions reach toxic levels they are excreted through dedicated transporters [13,14]. Finally, many cells are able to produce or import so-called compatible solutes that may accumulate to high intracellular concentrations without severely affecting cellular processes [15–17]. Beyond balancing intracellular osmolality, certain compatible solutes contribute to protein stabilization [18,19] and interact with cytoplasmic membrane lipids [20,21].

The ability to accumulate compatible solutes is conserved across all kingdoms of life [16] and highlights the pivotal role of metabolism in cellular osmoregulation. Chemically, most known compatible solutes are either polyhydroxylated compounds such as sugars or polyols (e.g. mannitol, trehalose), amino acids (e.g. proline, glutamine), N-acetylated diaminoacids (e.g. N_δ_-acetyl-ornithine, N_ε_-acetyl-lysine), ectoines (e.g. ectoine, β-hydroxyectoine), betaines (e.g. glycine betaine, β-alanine betaine) or thetines (e.g. dimethylsulfoniopropionate) that commonly share the properties of being polar, highly water-soluble and carrying no net charge at physiological pH [22,23]. The usage of certain compatible solutes such as glycine betaine is conserved, whereas other compounds appear to be used only by few related species, for instance β-amino acids and derivatives in methanogenic archaea [16,22]. The accumulation and maintenance of large intracellular compatible solute pools constitutes a major investment of cellular resources in terms of carbon and energy. Consequently, the re-routing of metabolic flux through osmolyte-producing reactions is expected to interfere with other metabolic processes these resources are diverted from. Moreover, high extracellular osmolality affects metabolism in additional ways, for example through direct inhibition of certain metabolic enzymes by inorganic salts [24–26] or through pleiotropic effects related to growth rate reduction [27,28]. Yet, compared to the role of compatible solutes, these global metabolic responses remain poorly understood and have only been investigated in few species [10,29–32]. Furthermore, it remains largely unclear whether the metabolic phenotype of a species correlates with its ability to tolerate certain levels of osmotic stress, a relevant question with implications for biomedical and biotechnological research.

Here, we determined the global metabolic responses of fifteen phylogenetically distant species with a broad spectrum of tolerances to sustained hyperosmotic salt stress. Using nontargeted mass spectrometry [33], we measured semiquantitative levels of hundreds of metabolites that provide detailed insights into the salt-stress induced metabolic adaptations. We found global metabolic responses to be predominantly species-specific, but individual metabolites showing characteristically similar responses in phylogenetically closely related organisms. Finally, we demonstrate that the responses of intermediates of certain metabolic pathways, including central carbon metabolism and heme biosynthesis, consistently correlated with salt tolerance, and discuss how these pathways might be linked to salt tolerance.

## Results

### Species differ considerably in salt tolerance

**Fig 1:**
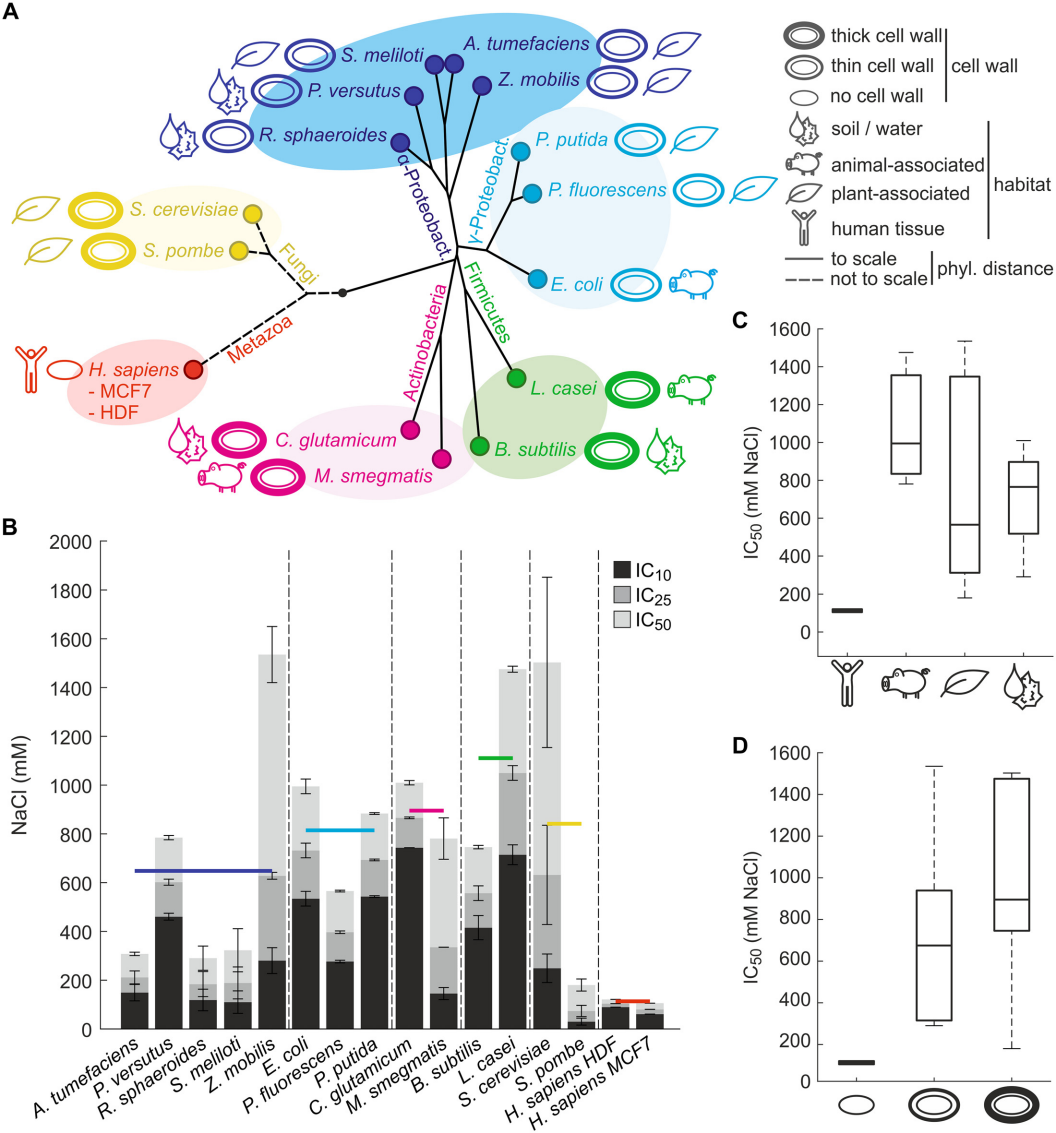
Analysis of salt tolerance in fifteen diverse species. (A) Phylogenetic tree of analyzed species. Jukes-Cantor distances between bacteria are drawn to scale based on aligned 16S small ribosomal subunit RNA sequences. Distances between eukaryotes are not drawn to scale for visualization purposes. Organisms are colored based on taxonomic classification, and cell wall strengths and typical habitats are indicated. Further information about strains and cell lines is provided in S1 Table. (B) Sustained hyperosmotic salt tolerance based on growth inhibition experiments. Salt tolerance is expressed as mean and standard deviation (n = 2) of concentrations inhibiting growth rates by 10% (IC_10_), 25% (IC_25_) and 50% (IC_50_) compared to unstressed conditions. Species are grouped according to taxonomic classification, and the colored horizontal bars indicate the average IC_50_ of each taxonomic group. (C) Comparison of salt tolerance between species colonizing different habitats. (D) Comparison of salt tolerance between species with different cell wall strengths. Differences between groups in panels B to D were not statistically significant (P > 0.05, unpaired two-tailed *t*-tests) for all comparisons except those with human cell lines.

Fourteen diverse pro- and eukaryotic microbial species (eight Gram-negative bacteria, three Gram-positive bacteria, one acid-fast bacterium and two yeasts) were selected based on their status as model organisms and common use in industry, thereby ensuring broad practical and academic relevance of our findings. The selected bacteria include the Gram-negative model bacterium *Escherichia coli*, the Gram-positive model bacterium *Bacillus subtilis* commonly employed as a generally regarded as safe industrial production organism, the Gram-negative bacterium *Zymomonas mobilis* that efficiently converts renewable feedstock into biofuels such as ethanol, as well as the Gram-positive acid-fast bacterium *Mycobacterium smegmatis*, a non-pathogenic and experimentally tractable proxy for the tuberculosis agent *M*. *tuberculosis*. Beside the model yeast *Saccharomyces cerevisiae* that is also widely used as production organism in industry and in food processing, we also selected the fission yeast *Schizosaccharomyces pombe* that is commonly used to study fundamental aspects of symmetric eukaryotic cell division. Additionally, we selected two human cell lines (primary dermal fibroblasts and immortalized breast adenocarcinoma cells) to assess responses in healthy and cancerous mammalian cells (Fig 1A and S1 Table).

To quantify species salt tolerance we performed growth inhibition assays. From constructed dose-response curves (S1 Fig) we determined the salt concentrations inhibiting growth rates by 10% (IC_10_), 25% (IC_25_) or 50% (IC_50_) compared to non-inhibitory conditions (Fig 1B and S2 Table). The observed salt tolerances varied considerably, with IC_50_ values ranging from 150 mM in the human MCF7 cell line to 1,500 mM NaCl in *Z*. *mobilis*. The taxonomic classification of organisms did not correlate with salt tolerance (Fig 1B) except for the two human cell lines that were particularly sensitive, presumably because osmolality in human body tissues generally is tightly regulated [34]. Natural microbe habitats also had no effect on their salt tolerance (Fig 1C). Although animal-associated microbes were slightly more tolerant than those free-living or associated with plants, the effect was not statistically significant (P ≥ 0.05, unpaired two-tailed *t*-tests). Microbes with thick cell walls were on average slightly more tolerant than thin-walled species, but this difference was again not statistically significant (P ≥ 0.05, unpaired two-tailed *t*-tests; Fig 1D), which is consistent with the main role of the cell wall of preventing cell lysis under hypoosmotic stress. The observation that neither taxonomic classification, natural habitat nor cell envelope structure were sufficient to explain the hyperosmotic salt stress tolerance indicates that the underlying molecular mechanisms are more deeply rooted in cellular physiology.

### Measuring metabolome responses to hyperosmotic salt stress

To obtain deeper insights into the physiological adaptations to sustained hyperosmotic salt stress, we measured intracellular metabolite levels. To ensure comparable perceived stress severities despite the considerable variations in salt tolerance, we subjected each species to salt concentrations inhibiting their growth rate by either 0% (control), 10% (low stress), 25% (moderate stress) or 50% (high stress) (S2 Table), and harvested cultures during exponential growth at an optical density at 600 nm of 1.0 (microbes) or at 50% confluency (human cell lines). Intracellular metabolites were extracted first with a polar and subsequently with an unpolar solvent to capture both hydrophilic and hydrophobic compounds, and the extracts were separately analyzed using nontargeted flow-injection time-of-flight mass spectrometry [33]. After spectral data processing and merging, we detected 17,727 distinct *m*/*z* features (ions). For each organism we retrieved species-associated metabolites from the Kyoto Encyclopedia of Genes and Genomes (KEGG) database [35] to annotate ions based on accurate mass. For metabolites with a logarithmic octanol/water partitioning coefficient (logP_O/W_) ≤ 0 data from polar and for metabolites with logP_O/W_ > 0 data from nonpolar extracts were used. Depending on the species we detected between 662 and 1,248 potential metabolites (S1 Data), with the method-inherent limitation of not being able to distinguish between isobaric compounds. This broad metabolome coverage provides detailed insights into the global metabolic responses to hyperosmotic salt stress.

### Known relative osmoprotectant levels and their stress-induced accumulation

**Fig 2:**
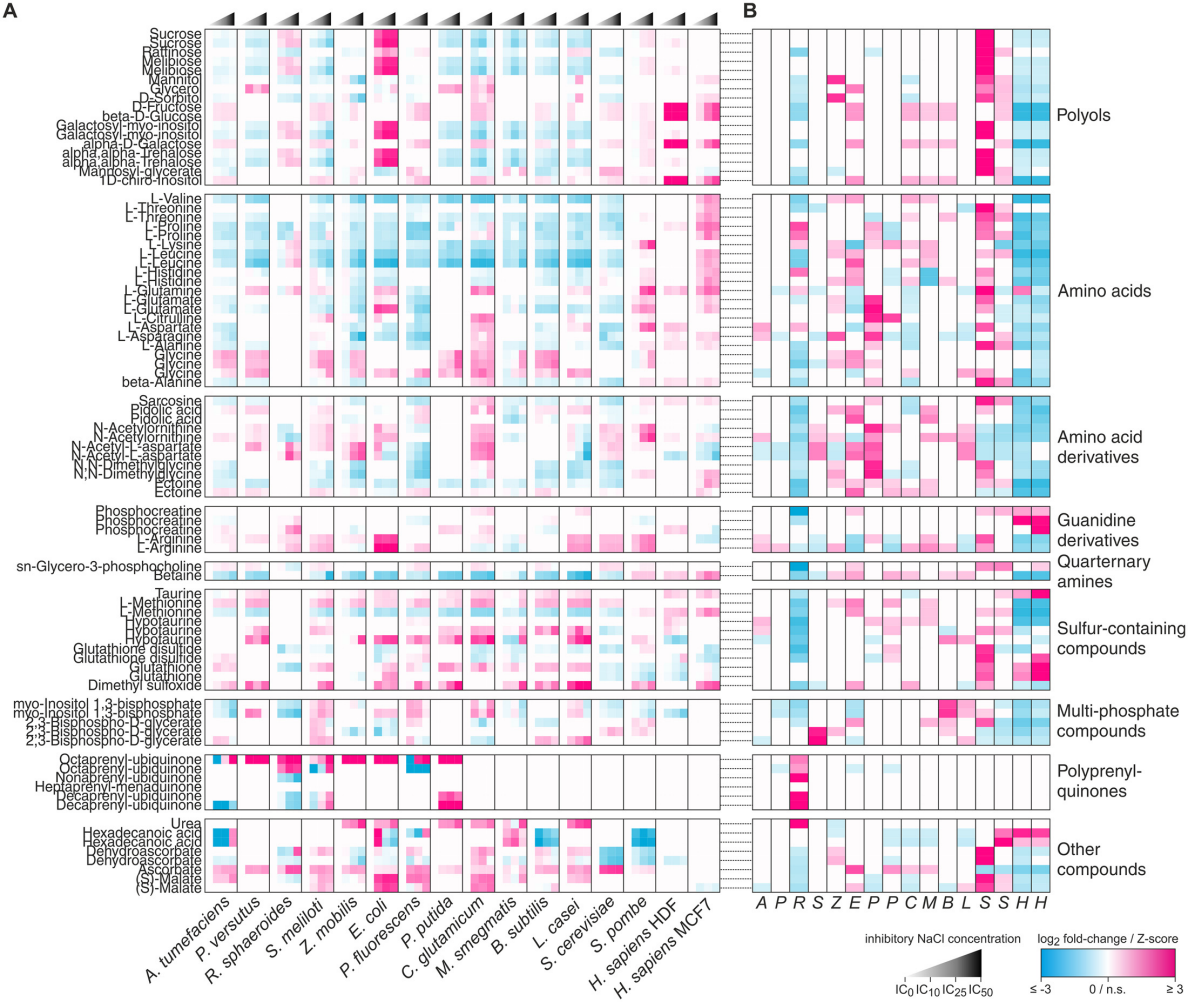
Responses of known osmoprotectants to sustained hyperosmotic salt stress. (A) Fold changes relative to unstressed control conditions of 54 detected metabolites with confirmed osmoprotective activity listed in the DEOP database [36], as well as of additional relevant polyprenyl quinones. For each species, only osmoprotectants showing a significant response (|log_2_ fold-change| ≥ 1, *P* < 0.05, multiple-testing corrected two-sided unpaired *t*-tests) in at least one stress intensity were considered. Duplicate names represent different ions of the same metabolite because accurate mass annotation was ambiguous; refer to S1 Data for complete annotations. Osmoprotectants were grouped based on their main chemical characteristics. (B) Baseline abundances of osmoprotectants at IC_0_ (unstressed control condition). Data are represented as Z-scores to compare relative abundances between species. Small Z-scores (|Z| < 1) are not colored.

To systematically assess the use of known compatible solutes and other osmoprotectants across all species, we analyzed the responses of 54 detected metabolites with confirmed osmoprotective activity listed in the DEOP database [36] by computing fold-changes relative to unstressed control conditions. While literature reports suggest that only few compatible solutes are used per species (S1 Table), we typically observed a complex pattern of dozens of potentially accumulating osmoprotectants (Fig 2A). We emphasize that our analytical platform allows only to determine relative metabolite levels, but not their absolute concentrations. Hence, it is possible that some of the detected osmoprotectants reach insufficient absolute concentrations to exert substantial impact on osmotolerance on their own. Nevertheless, the cumulative effects of the large number of observed osmoprotectants is likely to contribute at least partially to osmotolerance, thereby complementing and modulating the effects of established major compatible solutes. For instance, in *E*. *coli* we not only observed accumulation of its known compatible solutes trehalose and glutamate [37,38] and the membrane-stabilizing isoprenoid ubiquinone-8 [10], but also increasing levels of other osmoprotectants such as hypotaurine, arginine, malate or N-acetylornithine. Similarly, *C*. *glutamicum* not only accumulated proline, but also other amino acids and derivatives including N-acetylaspartate, N-acetylornithine, N,N-dimethylglycine or glycine. Other examples include the accumulation of disaccharides and N-acetylaspartate in *R*. *sphaeroides* or hypotaurine and glycerol in *P*. *versutus*. Finally, also the human MCF7 cell line accumulated various amino acids and ectoine in addition to the known compatible solutes inositol, betaine, taurine and sorbitol.

Some osmoprotectants accumulated in several species, whereas others appeared to be species-specific, in particular in the two human cell lines. For example, the amino acids threonine, proline, lysine, leucine and histidine mostly decreased in microbes but increased in the MCF7 cell line. Similarly, accumulation of monosaccharides and related compounds such as hexoses, inositol or sorbitol was also mostly observed in the human cell lines. Furthermore, the Gram-negative proteobacteria were the only group of species found to accumulate polyprenyl quinones, hydrophobic compounds presumably increasing the stability of the cytoplasmic membrane [10,39], suggesting membrane stabilization to be mainly important for bacteria with thin cell walls. Conversely, other osmoprotectants were more widely used. We noticed that these included many simple nitrogen- or sulfur-containing compounds such as urea, hypotaurine or glycine that can be synthesized relatively easily either *de novo* or from precursors in the culture medium without requiring the expression of enzymes belonging to complex pathways. Betaine, a compatible solute that is known to be used by species of all domains of life [16], was not among these generally accumulating osmoprotectants, presumably because many species rely on its import from the extracellular medium that in our case did not contain betaine or its precursor choline in sufficient amounts.

In principle osmotolerance may also be achieved by naturally high levels of intracellular osmoprotectant. To compare basal abundances of the 54 known osmoprotectants between species, we calculated Z-scores of these compounds in the unstressed control condition (Fig 2B). Indeed, we observed that several species had high levels of certain osmoprotectants even in absence of salt stress. For instance, *R*. *sphaeroides* had relatively high levels of polyprenyl quinones, indicating a particular relevance of membrane stabilization in this organism regardless of applied stress, indeed one reason why this organism has been deemed a suitable host for ubiquinone-10 production [40]. Similarly, high levels of the polyols sorbitol and mannitol in *Z*. *mobilis* and several amino acids and derivatives in *P*. *fluorescens* indicated these compounds to convey passive basal osmoprotection to these species. Most strikingly were the relatively high levels of a large number of diverse osmoprotectants, including polyols, amino acids and the redox-active compounds glutathione, glutathione disulfide and dehydroascorbate in the yeast *S*. *cerevisiae*. Our data therefore suggests that in *S*. *cerevisiae*, aside from the well-established accumulation of glycerol the naturally high levels of osmoprotectants and stress-alleviating compounds additionally contribute to salt stress tolerance, consistent with previous studies showing that in *S*. *cerevisiae* absolute levels of known osmoprotectants accounted for 80–90% of the covered metabolome [41,42] compared to 70% in *E*. *coli* [43] (S2 Fig). Thus, maintaining generically high osmoprotectant levels may be a complementary strategy to salt-stress induced compatible solute accumulation that would require less additional protein synthesis, less metabolic rearrangements and less complex regulation.

### Global metabolic salt stress responses are predominantly species-specific

**Fig 3:**
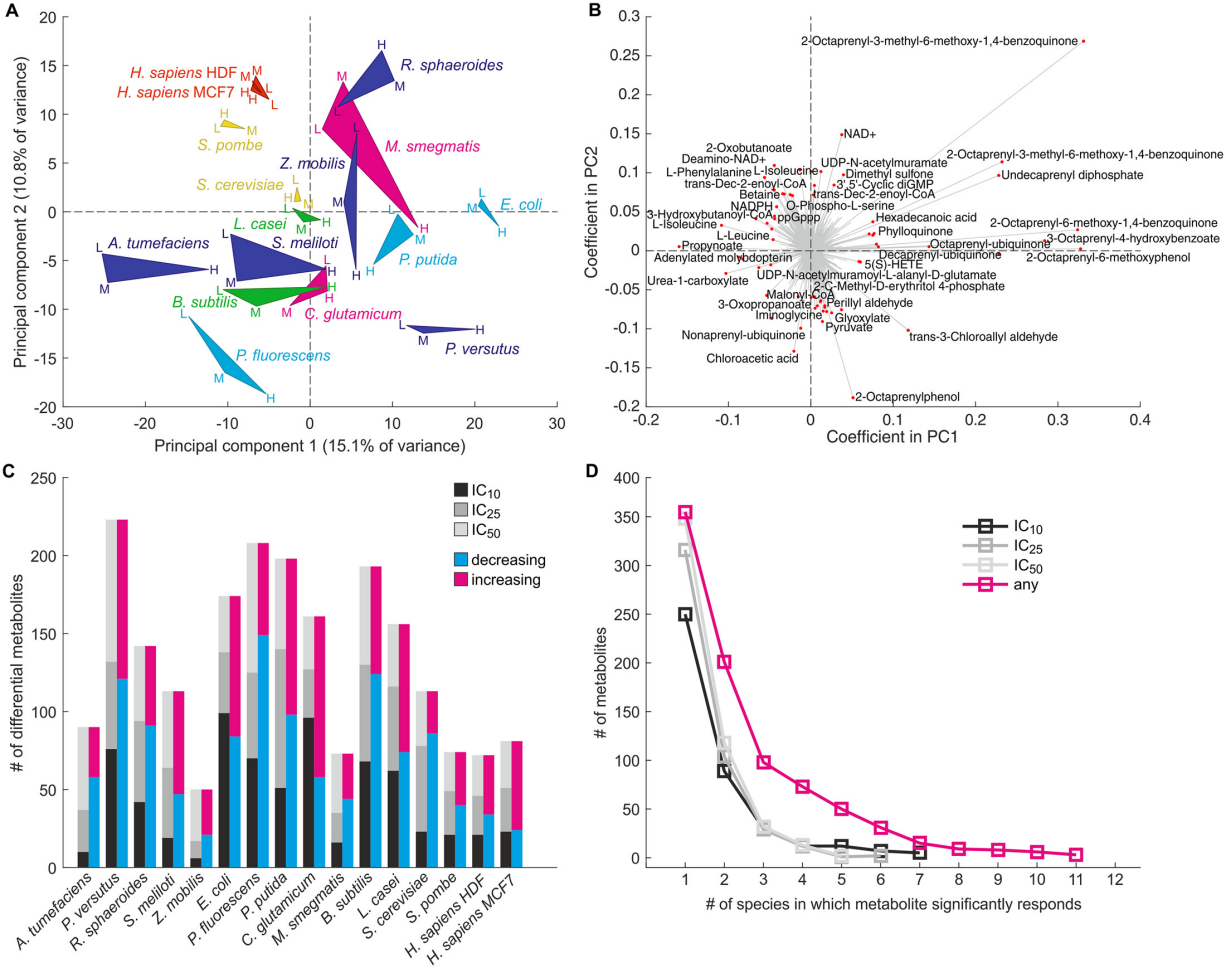
Hyperosmotic salt stress elicits complex and predominantly species-specific global metabolic responses. (A) Principal component analysis (PCA) was performed based on log_2_ metabolite ion fold-changes upon low (IC_10_, L), medium (IC_25_, M) or high (IC_50_, H) salt stress relative to unstressed controls. For each species the three stress intensity points are connected by triangular patches for visualization purposes. Patches and labels are colored according to taxonomic classification as defined in Fig 1A. (B) Loading plot of metabolites underlying the PCA shown in panel A. Selected metabolites with large coefficients are highlighted. Note that metabolite annotations are based on accurate mass and can be ambiguous; refer to S1 Data for complete annotations. (C) Numbers of strongly and significantly responding metabolites in each analyzed species, grouped either by the lowest stress intensity under which a change was observed (gray bars) or by change direction (magenta and blue bars). (D) Histogram of the number of species in which metabolite ions were affected by the individual salt stress intensities (black, dark gray and light gray curves) or by at least one stress intensity (magenta curve).

To investigate the global metabolic responses to hyperosmotic salt stress more deeply beyond the accumulation of osmoprotectants, we determined the fold-changes of all detected metabolite ions relative to the isosmotic control condition (S1 Data). In a principal component analysis species did not segregate into distinct clusters (Fig 3A and 3B), indicating that global metabolic responses were not dominated by specific inter-species relationships. The number of differential metabolites, again defined as having a |log_2_ fold-change| ≥ 1 and a P-value < 0.05 (unpaired two-tailed *t*-tests, multiple-testing corrected) in at least one salt stress intensity, varied considerably between species (Fig 3C). On average 133 metabolites were affected with the extremes of 50 and 223 responding metabolites in *Z*. *mobilis* and *P*. *versutus*, respectively. As expected, the number of differential metabolites increased with increasing salt concentrations in all species, confirming that higher cellular stress was reflected in the metabolome, presumably mainly through pleiotropic effects caused by growth rate reduction or changes in cell size. In most species the majority of differential metabolites were depleted, possibly because stressed cells devoted more resources to osmoprotection at the expense of other biosynthetic tasks. The complex responses were largely species-specific because most metabolites were only affected in one or two species (Fig 3D).

**Fig 4:**
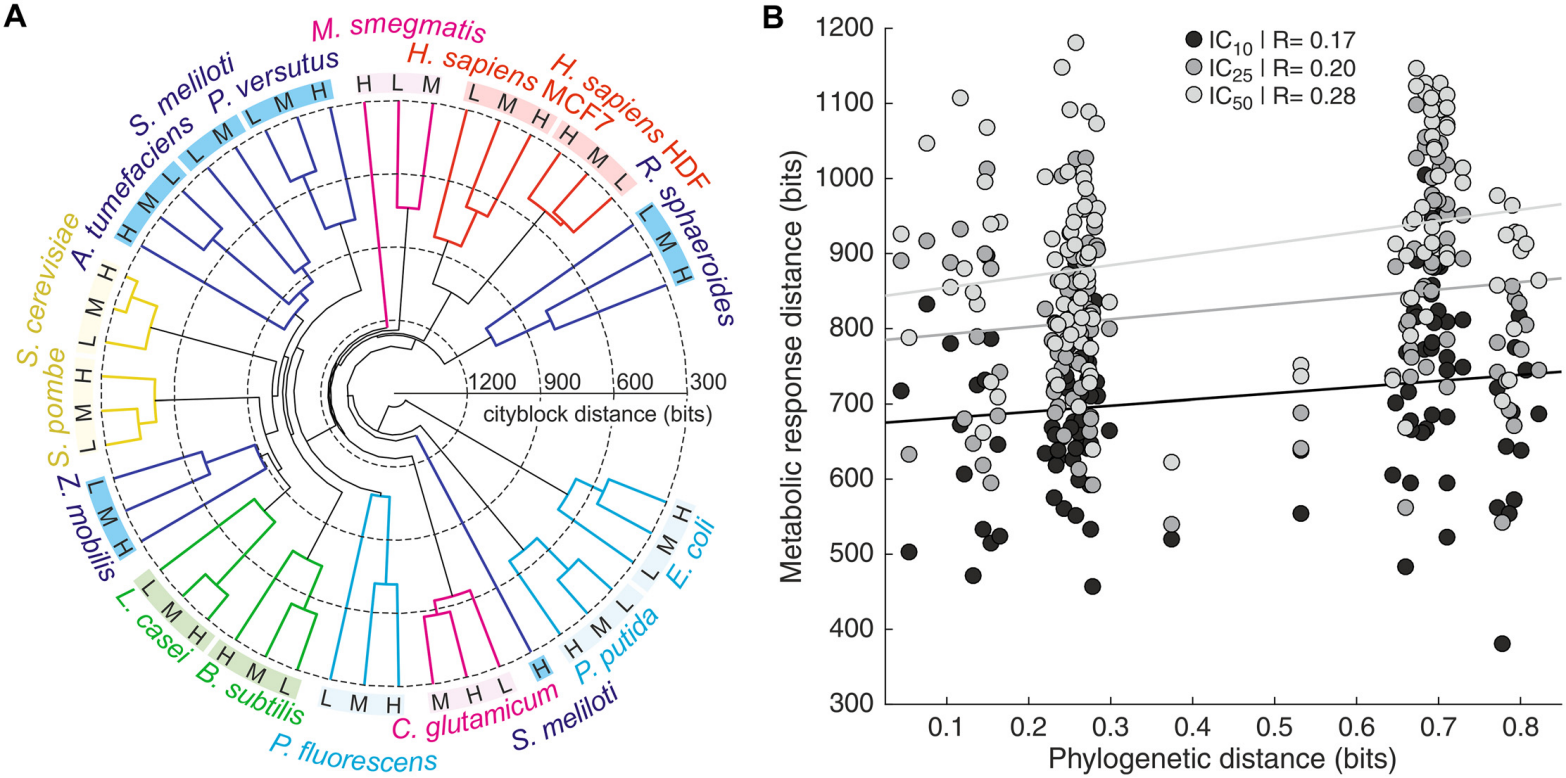
Phylogenetic relationship between species is insufficient to explain differences in metabolic salt stress responses. (A) Cladogram of analyzed species based on metabolite ion log_2_ fold-changes upon exposure to low (IC_10_, L), medium (IC_25_, M) and high (IC_50_, H) salt stress relative to unstressed controls (IC_0_). Pairwise distances between samples were calculated using the Cityblock metric. Species labels are colored according to taxonomic classification as defined in Fig 1A. (B) Correlation of pairwise distances between species based on metabolic salt stress responses with phylogenetic distances. Distances between metabolic responses to different salt stress severities were calculated based on metabolite ion log_2_ fold-changes relative to IC_0_ using the Cityblock metric, and phylogenetic distances based on the aligned small ribosomal subunit RNA sequences using the Jukes-Cantor measure. R indicates Pearson’s correlation coefficient.

To test whether differences between metabolic responses reflected the phylogenetic relationship of the analyzed species, we constructed a cladogram from the determined metabolite fold-changes (Fig 4A). Reassuringly, samples of the three salt stress intensities clustered together for all but one species, demonstrating that the metabolic responses to varying stress intensities within a species were more similar to each other than to responses of different organisms. Intriguingly, the cladogram did not resemble the phylogenetic tree (compare with Fig 1A) aside from the two yeasts, the two human cell lines and the two firmicutes each grouping together. For instance, the two actinobacteria were separated from each other, as were the five proteobacteria. The major difference was the absence of a clear separation of pro- and eukaryotes, a hallmark of phylogeny. To ascertain this observation, we systematically correlated all pairwise metabolic response distances with the phylogenetic distances between the analyzed species (Fig 4B). Indeed, although correlations were positive and statistically significant at higher stress intensities (P = 0.062 at IC_10_, P = 0.031 at IC_25_ and P = 0.002 at IC_50_, modified Student’s *t*-tests) they were weak (Pearson’s R between 0.17 and 0.28), confirming that phylogenetic relationship was not a major determinant of the metabolic salt stress response. Similarly, neither natural habitat, cell envelope structure, nor salt tolerance of the species could explain the global metabolic response (S3 Fig).

**Fig 5:**
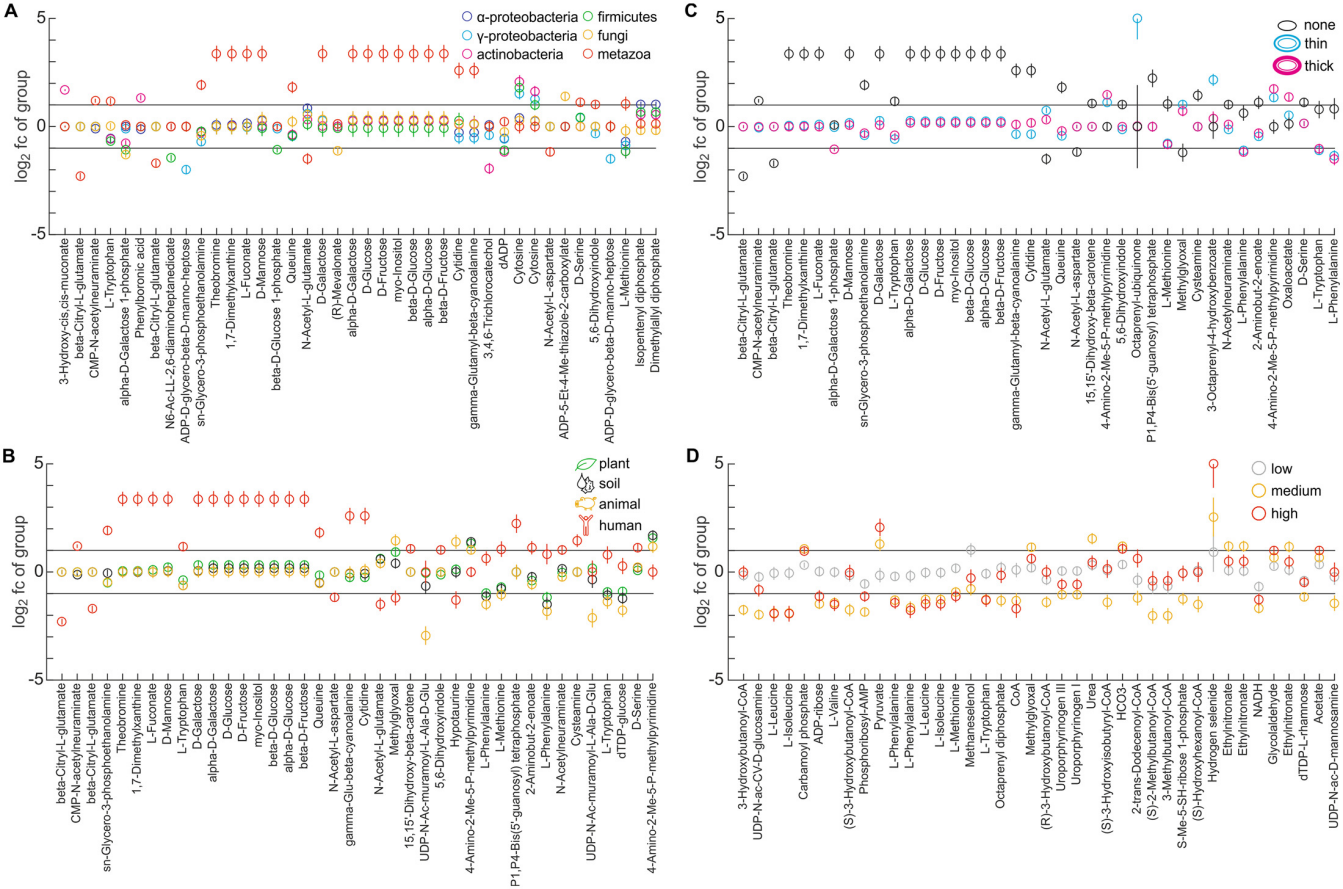
Individual metabolite responses are influenced by species taxonomy, habitat, cell wall thickness and salt tolerance. Four-way analysis of variance (ANOVA) was performed on log_2_ metabolite fold-changes in the different species upon high salt stress (IC_50_). Shown are the mean and standard errors of the 40 most significant metabolites of each factor (all with ANOVA *P* < 0.01 and |log_2_ fold-change| > 1 in at least one group). Metabolites are sorted from left to right by ascending *P*-value. (A) Grouping of species according to taxonomic classification. (B) Grouping according to habitat. (C) Grouping according to cell wall thickness. (D) Grouping according to salt tolerance (IC_50_ < 500 mM NaCl = low; 500 ≤ IC_50_ ≤ 1,000 mM = medium; IC_50_ > 1,000 mM = high). Note that metabolite annotations are based on accurate mass and can be ambiguous; refer to S1 Data for complete annotations.

To test whether phylogeny or other factors influence subsets of the metabolome changes, we systematically compare the responses of individual metabolites by a four-way analysis of variance (ANOVA). Indeed, we found 137 metabolites that were able to strongly and significantly (average log_2_ fold-change in most distinct group > 1, ANOVA P-value < 0.01) explain the differences between the groups of at least one factor. For 97 of these metabolites the variance depended on taxonomic grouping (Fig 5A). For instance, the two human cell lines specifically accumulated hexoses which was not observed in any of the microbes, consistent with the previously reported accumulation of inositol in human renal cells [17]. Another human-specific response was the depletion of β-citryl-L-glutamate, an iron-chelating compound that was recently shown to maintain mitochondrial oxidative phosphorylation by preventing the inactivation of aconitase upon oxidative stress [44], which might explain the observed hexose build-up by blocking the tricarboxylic acid cycle flux. Other examples for taxonomy-dependent metabolite responses are the accumulation of cytosine by *Actinobacteria*, *Firmicutes* and *γ-Proteobacteria* as well as the *γ-Proteobacteria*-specific depletion of the lipopolysaccharide precursor ADP-L-glycero-β-D-manno-heptose, demonstrating that the phylogenetic relationship among species does influence the metabolic salt stress response, albeit only at the level of individual metabolites. Similarly, 77 and 73 significantly different metabolites were observed when considering natural habitat or cell wall structure (Fig 5B and 5C), for instance the depletion of the cell wall precursor UDP-N-acetylmuramoyl-L-alanine-D-glutamate in animal-associated bacteria or the accumulation of ubiquinone-8 and its precursor 3-octaprenyl-4-hydroxybenzoate in thin-walled gram-negative bacteria. Although these factors obviously are partially interdependent with taxonomy, our data nevertheless suggest that natural habitat and cell wall structure may contribute to the response of specific metabolites to salt stress.

The final, and perhaps most intriguing, factor we considered was salt tolerance, which we found to be largely independent of taxonomic classification, natural habitat or cell wall structure (Fig 1B). Indeed, we observed 43 significantly different metabolites among species with different levels of salt tolerance (Fig 5D). Most of these characteristic metabolites specifically depleted in salt-tolerant species while only few accumulated, consistent with our previous analysis that indicated that the accumulation of individual osmoprotectants did not depend on salt tolerance (Fig 2A). One large group of metabolites that consistently depleted in salt-tolerant species were various amino acids including leucine, tryptophan or methionine, implying that salt-tolerant species may either reduce their biosynthesis to re-direct their resources towards stress responses or conversely use these compounds as precursors for other processes involved in osmoprotection, such as the synthesis of osmoprotectants as we will discuss below. A second group of metabolites were various CoA thioesters and CoA itself that also depleted more strongly in salt-tolerant species. The depletion of CoA and derivatives has been previously observed in salt-stressed *E*. *coli* cells [10,45] but its functional role is unclear, in particular since higher levels of CoA were found to actually improve salt tolerance in plants by altering lipid metabolism [46]. Thus, although the global metabolic responses were mainly species-specific, the response of individual metabolites consistently depended phylogeny, habitat, cell wall structure and salt tolerance.

### Responses of several metabolites and pathways correlate with salt tolerance

**Fig 6:**
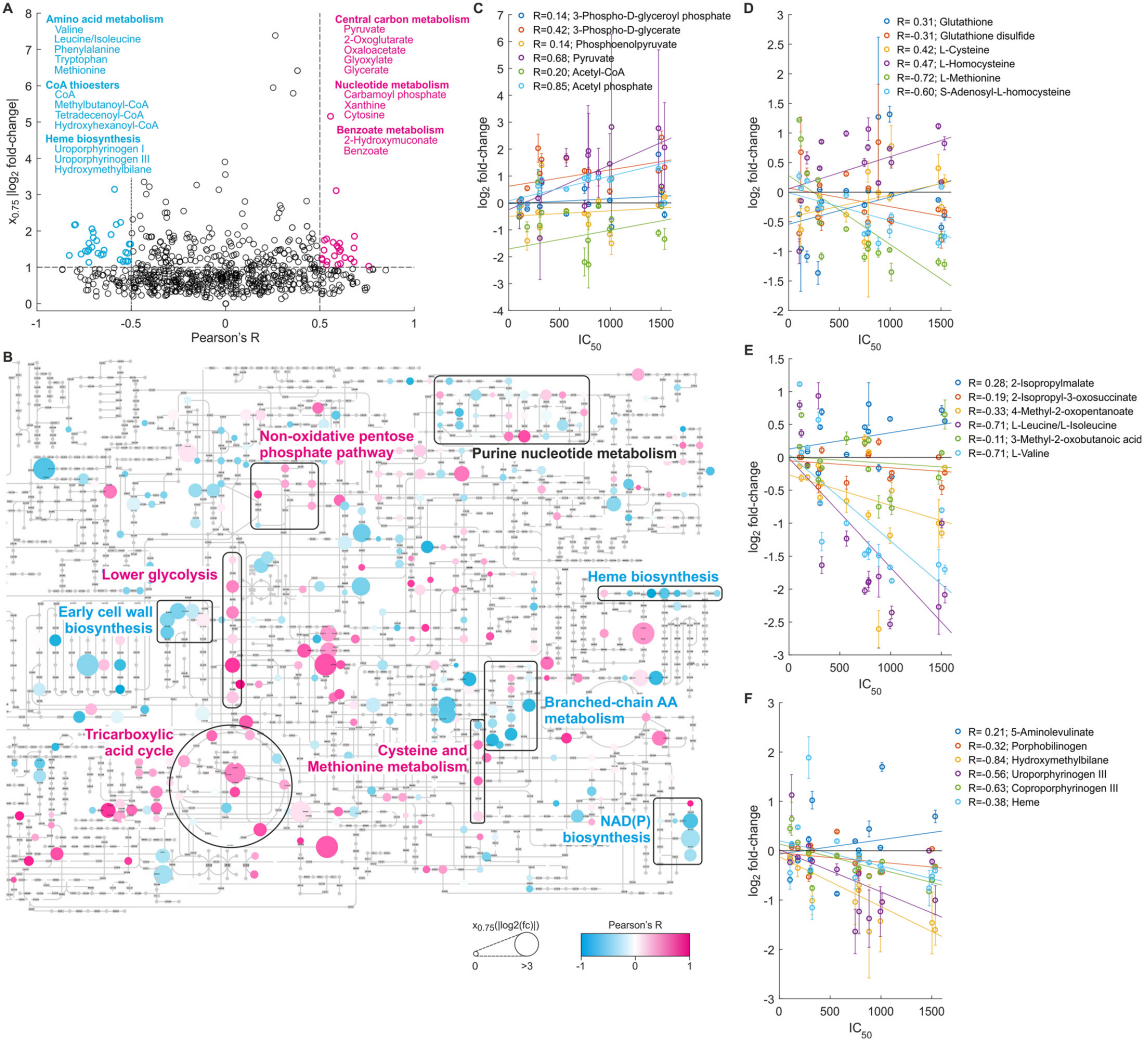
Correlation analysis of metabolite responses with salt tolerance. (A) Correlation of metabolite fold-changes in response to strong salt stress with IC_50_ values of the different organisms was assessed by Pearson’s correlation coefficient R. For each metabolite, the upper quartile x_0.75_ of absolute log_2_ fold-changes across all species was plotted against R. Only metabolites detected in more than 10 species were considered. Metabolites with |R| > 0.5 and x_0.75_ > 1 are highlighted in blue (anticorrelating metabolites) and pink (correlating metabolites), and the names of representative compounds are listed. The full correlation data is provided in S2 Data. (B) Visualization of metabolites correlating with salt tolerance on the KEGG metabolic pathway map using PathwayProjector [47]. Color intensity of metabolites indicates strength of positive (pink) or negative (blue) correlation, and size indicates x_0.75_. Key pathways are highlighted and labeled. (C) Correlation of fold-change with salt tolerance for selected compounds in lower glycolysis; (D) in cysteine and methionine metabolism; (E) in branched-chain amino acid metabolism; and (F) in heme biosynthesis. In panels C to F mean and standard deviation of four (microbes) or three (human cell lines) replicates are shown. Note that metabolite annotations are based on accurate mass and can be ambiguous; refer to S1 Data for complete annotations.

So far, we identified metabolites potentially associated with salt tolerance based on their specific responses in salt-tolerant and salt-sensitive species. Yet, salt tolerance is not a discrete phenotype but rather a continuous property influenced by the interplay of various cellular and environmental factors, which likely explains the diverse metabolic responses we observed even among comparably tolerant cells. We thus performed a correlation analysis to identify metabolites of which the response gradually increased or decreased with increasing IC_50_ values of analyzed species (Fig 6A and S2 Data). In total the responses of 36 metabolites anticorrelated (Pearson’s R < -0.5) and 25 metabolites correlated (R > 0.5) well with salt tolerance and additionally showed an absolute log_2_ fold-change > 1 in at least 25% of the species, indicating a consistently strong response to salt stress. Among the anticorrelating metabolites were the branched-chain amino acids valine, leucine and isoleucine, the aromatic amino acids phenylalanine and tryptophan, the sulfur-containing amino acid methionine, various CoA thioesters and CoA itself, as well as several intermediates of heme biosynthesis. Among the correlating metabolites were a considerable number of compounds of central carbon metabolism including pyruvate and 2-oxoglutarate.

To obtain a systematic overview in which pathways these correlating metabolites were involved, we visualized them on the KEGG global metabolic pathway map using PathwayProjector (Fig 6B) [47]. While some correlating metabolites were seemingly disconnected from neighboring compounds, many others clustered in canonical pathways. Strikingly, the responses of virtually all intermediates of the lower glycolytic pathway and many compounds in the tricarboxylic acid cycle and the pentose phosphate pathway showed a positive correlation with salt tolerance (Fig 6C). While our data do not establish any causality, this result would be consistent with the hypothesis of increased energy production and redox cofactor regeneration in salt-tolerant species, potentially enabling these species to allocate more energy and building blocks to the biosynthesis of osmoprotectants, or to mitigate adverse secondary effects by, for example, more efficient clearance of reactive oxygen species (ROS) enabled by NADPH-dependent enzymes such as glutathione reductase [48]. Moreover, we observed a positive correlation of several intermediates in the biosynthesis of methionine and cysteine (Fig 6D), the latter containing the active moiety and being a direct precursor of the well-known ROS scavenger glutathione. Likewise, levels of late intermediates and the end-products leucine, isoleucine and valine in branched-chain amino acid metabolism decreased more strongly in salt-tolerant organisms (Fig 6E). A potential role of this strong depletion could be that branched-chain amino acids are used as ammonium donors in transamination reactions with 2-oxoglutarate to produce higher cellular levels of the main osmoprotective amino acids glutamate and proline. This route leading to osmolyte accumulation has indeed been observed in the bacterium *Rhizobium meliloti* in which exogenous addition of leucine stimulated glutamate biosynthesis under osmotic stress [49], as well as in plants in which drought induced the expression of branched-chain amino acid transferases [50,51]. Our data suggest this mechanism to be widely conserved and quantitatively linked to salt tolerance. We did not observe a strong accumulation of glutamate itself in most species, but as intracellular levels of glutamate in unstressed cells generally are considerably higher than those of other amino acids this would not necessarily be expected. Finally, we highlight heme biosynthesis whose intermediates strongly anticorrelated with salt tolerance. (Fig 6F). Heme is a widely conserved iron-chelating cyclic tetrapyrrole that is a prosthetic group of proteins involved in the respiratory electron transport chain and many other cellular processes [52], and we below discuss several hypotheses how heme depletion could be linked to salt tolerance.

## Discussion

In this work we employed nontargeted metabolomics to investigated the global metabolic responses of twelve diverse bacteria, two yeasts and two human cell lines exposed to hyperosmotic salt stress. We observed that in all organisms salt stress affected the abundances of dozens of metabolites throughout their metabolic networks, but surprisingly most metabolites responded only in one or two species. This highly species-specific global metabolic response at first seems unexpected, since the ubiquitous occurrence of salt stress suggests it must have been encountered already during early stages of life, and one might therefore have expected a more conserved response. Yet, it is well-known that salt tolerance itself can considerably vary sometimes even among species of the same genus [53], as can the usage of compatible solutes [22,54]. Moreover, species belonging to the same taxonomic groups frequently have fundamentally different metabolic phenotypes and lifestyles and consequently are likely to have evolved response mechanisms tailored to their particular needs. In this light, our observations support the notion that phylogeny is not a major determinant of how cells respond to salt stress.

Which other factors, then, can explain these distinct metabolic responses? An obvious hypothesis we tested was that similar levels of salt tolerance would coincide with similar changes in metabolism. Yet, we found no evidence that the observed global metabolic responses depended on how much salt the individual species were able to tolerate. One possibility is that species with higher salt tolerance have higher basal levels of osmoprotectants already in unstressed conditions, a phenotype that we indeed observed in our study for some salt-tolerant organisms, in particular *S*. *cerevisiae*. A second possibility arises from differences in metabolic regulation between different species, meaning that the extent of cross-talk between salt stress response pathways and other cellular processes is likely to vary. A third possibility is that certain enzymes are differentially influenced by salt, leading to species-specific responses of metabolites in their metabolic neighborhood. We furthermore considered the role of the natural habitat of analyzed organisms, since severity and frequency of experienced salt stress, the availability of osmoprotectants as well as the co-occurrence of other perturbations and stimuli are likely to shape the global metabolic salt stress response through evolutionary selection. However, our analyses did not provide evidence that certain habitats would pre-dispose organisms to exhibit particular metabolic responses. One possible reason is that the habitat definitions we applied were rather coarse-grained and that the investigated species within individual habitats were highly diverse, meaning that our dataset may lack the statistical power required to reveal potential associations, which could be addressed in the future by a deeper coverage of species colonizing similar habitats. Another reason could be that we chose to cultivate all species in similar experimental conditions, thereby potentially masking natural environmental influences. Yet, disentangling environmental from salt-triggered metabolic effects would have been demanding and likely non-conclusive, at least for the diverse selected species.

An unprecedented analysis made possible by our global metabolomics approach was the correlation of the response magnitudes of individual metabolites and pathways with the salt tolerance of analyzes species. Whereas the responses of pathway intermediates were too weak to be statistically significant in single species on their own, the correlation of response magnitude with salt tolerance over a wide range of species does suggest that the implication of these pathways in the metabolic response to osmotic stress is conserved. Among the pathways with consistent correlations of multiple intermediates were central carbon metabolism and energy generation, branched-chain amino acid metabolism and heme biosynthesis. A more active central carbon metabolism might provide salt-tolerant organisms with the necessary energy and building blocks to fuel processes conveying salt tolerance, such as the biosynthesis of compatible solutes. Furthermore, reduced levels of branched-chain amino acids may indicate their increased consumption in transamination reactions fueling the accumulation of the osmoprotectant glutamate.

Finally, we propose three not mutually exclusive hypotheses how a depletion of heme biosynthesis intermediates could be linked to salt tolerance. First, we note that in most species the committed steps of heme biosynthesis are the reactions converting glutamate via glutamyl-tRNA^Glu^ and glutamate semialdehyde to 5-aminolevulinate [52]. The accumulation of glutamate as osmoprotectant may require cells to reduce glutamate utilization by competing processes such as heme biosynthesis, and organisms doing so more thoroughly would be able to maintain higher intracellular glutamate concentrations. A second possibility arises from a previously reported connection between iron homeostasis and osmotic stress in the halophilic bacterium *Chromohalobacter salexigens* which was found to actively down-regulate iron demand and uptake when challenged by high salt concentrations [55]. Intracellular iron catalyzes the cleavage of hydrogen peroxide to hydroxyl and hydroperoxyl radicals in the Fenton reaction, a major source of ROS and oxidative cellular damage [56], and consequently lower heme-bound iron levels may reduce oxidative stress which can follow from osmotic stress [57,58]. Thus, a reason why certain species tolerate higher salt concentrations may be that they accumulate less ROS. The third and final hypothesis is that heme biosynthesis is reduced because the pathway is costly as it involves up to ten different enzymes and numerous complex cofactors including pyridoxal 5’-phosphate, tRNA, flavins and S-adenosyl-L-methionine [52]. Hence, species that more strongly reduced the copy numbers of enzymes involved in heme biosynthesis would have more resources available for osmoprotection. Yet, based on our data we cannot distinguish whether indeed transcriptional or translational regulation was responsible for salt-dependent changing levels of heme precursors, or whether other regulatory mechanisms not affecting protein levels, such as posttranslational regulation, were involved, and it will be interesting to probe these hypotheses in future investigations.

To our knowledge, the dataset we obtained is the so far most comprehensive compendium of salt stress-induced metabolic responses across a panel of such diverse species. Consequently, our data enable the contemplation at unprecedented depth not only of canonical responses such as the accumulation of compatible solutes [38], but also of numerous other metabolic alterations that might reflect intricate functional connections between different cellular processes. While identifying mechanisms underlying or causing these complex responses is challenging and clearly beyond the scope of this work, we above discussed several cases in which our data appears to corroborate and expand previously reported mechanisms. We project that enriching and complementing future analyses of the cellular salt stress response, possibly guided by computational models or the integration of orthogonal data [59], could become major applications of the resource we generated. Moreover, the diversity of analyzed species suggests this resource to be potentially relevant for various communities ranging from biotechnological to biomedical research. Altogether, our study has provided detailed insights into the pivotal role of metabolism for the cellular responses of diverse organisms to hyperosmotic salt stress, as well as a glimpse on the evolutionary and ecological determinants of salt tolerance.

## Material and Methods

### Strains and cell lines

Strains and cell lines used in this work and corresponding references are listed in S1 Table. Human cell lines were previously established and are commercially available (HDF cell line from Cell Applications Inc., San Diego, CA, USA, catalog no. 106-05n; MCF7 cell line from DSMZ, Braunschweig, Germany, catalog no. ACC115).

### Chemicals

All chemicals were purchased from Sigma Aldrich (St. Louis, MO, U.S.A.) at the highest available purity (typically >95%) unless indicated differently.

### Medium for bacterial cell cultures

The medium used for bacterial cell cultures was lysogeny broth (LB; 10 g/L Bacto tryptone, 5 g/L Bacto yeast extract, 5 g/L D-glucose) supplemented with NaCl as indicated.

### Medium for yeast cell cultures

The medium used for yeast cell cultures was YPD medium (10 g/L Bacto yeast extract, 20 g/L Bacto peptone, 20 g/L D-glucose) supplemented with NaCl as indicated.

### Human cell cultures

HDF cells were cultivated at 37°C at 5% CO_2_ in humidified atmosphere in Dulbecco’s modified Eagle medium (DMEM; Life Technologies) supplemented with 4.5 g/L D-glucose, 2 mM GlutaMAX, 10% fetal calf serum (PAA, Pasching, Austria), 100 U/mL penicillin/streptomycin and indicated amounts of NaCl and used at passage numbers four to six. MCF7 cells were cultivated under identical conditions in DMEM supplemented with nutrient mixture F-12 (Life Technologies), 4.5 g/L D-glucose, 2 mM GlutaMAX, 10% fetal bovine serum, 100 U/mL penicillin/streptomycin and indicated amounts of NaCl. Cells were maintained by splitting twice a week at a ratio of 1:6 at approximately 80% confluency by adding phosphate-buffered saline (PBS; Life Technologies) containing 0.25% trypsin for 2 min after washing with pre-warmed PBS.

### Salt tolerance analysis of bacterial cells

Precultures in 5 mL LB medium supplemented with 5 g/L NaCl were inoculated from -80°C glycerol stocks and incubated at 30°C in an orbital shaker at 300 r.p.m. for 24 h. From these precultures 5 μL were used to inoculate transparent flat-bottom 96-round-well plates containing 200 μL prewarmed LB medium per well supplemented with twelve different NaCl concentrations from 50 mM to 2,500 mM in duplicates. Cell growth of cultures shaken at 30°C was monitored in a TECAN Infinite M200 plate reading instrument (TECAN group) by measuring the absorbance at 600 nm every 10 min over a period of at least 24 h. From the resulting growth curves the maximum exponential growth rates were calculated, and IC_10_, IC_25_ and IC_50_ values were computed from sigmoidal curves fitted to maximum growth rates plotted against salt concentrations (S1 Fig).

### Salt tolerance analysis of yeasts

Salt tolerance analysis of yeasts was performed analogously to the analysis in bacteria, except that the medium was YPD, the 96-well plates used were transparent flat-bottom flower plates (M2P labs) and growth at 30°C was monitored by measuring culture turbidity in 5 min intervals over a period of at least 48 h using a BioLector (M2P labs) with ambient relative humidity set to 85%.

### Salt tolerance analysis of human cell lines

HDF and MCF7 cells were harvested at 80% confluency and seeded at a density of 10,000 cells/well in 96-well plates containing the respective media supplemented with 0 to 250 mM NaCl in six replicates per concentration. Cell numbers were determined after 24 h, 48 h and 72 h by washing with PBS, fixing for 15 min with 4% paraformaldehyde and staining nuclei with 1 μg/mL Hoechst 33342 dissolved in PBS. Hoechst fluorescence intensity per well (excitation wavelength 350 nm, emission wavelength 460 nm), correlating linearly with cell number as confirmed by dilution series (S4 Fig), was measured in a TECAN Infinite M200 plate reader (TECAN group) to quantify cell growth. Cell numbers after 72 h were plotted against salt concentrations, and IC_10_, IC_25_ and IC_50_ values were computed from a sigmoidal fit to these data (S1 Fig).

### Sample preparation for metabolomics

Microbes were grown in 1.5 mL of their respective media in 96-deepwell plates at 30°C shaking at 300 r.p.m. at indicated salt concentrations in quadruplicates until reaching an optical density at 600 nm of 1.0. A culture volume of 1 mL was then harvested by fast centrifugation (1 min at 13,000 g and 4°C) and quenched in liquid nitrogen until further processing. Metabolites from cell pellets were first extracted for 10 min at 85°C with 150 μL ethanol:water (70:30%-v/v) to capture polar compounds. After centrifugation (10 min at 13,000 g and 4°C) and collection of the supernatant, a second extraction with 150 μL chloroform:methanol (2:1%-v/v) was performed for 1 h at 4°C to capture nonpolar compounds such as lipids. Human cell lines were grown in 6-well culture dishes as described above in presence of indicated NaCl concentrations in triplicates until reaching 50% confluency. Cells were then rapidly washed twice with 75 mM ammonium carbonate at pH 7.4 and immersed in liquid nitrogen to quench metabolism. Metabolites were first extracted twice for 2 min at 85°C with 700 μL ethanol:water (70:30%-v/v) and, after collecting the supernatant, once for 1 h at 4°C with 1.5 mL chloroform:methanol (2:1%-v/v). Samples were stored at -80°C for at most two weeks prior to analysis.

### Flow-injection mass spectrometry

Metabolomics samples were analyzed by flow-injection time-of-flight MS [33] with an Agilent 6550 iFunnel QToF instrument (Agilent, Santa Clara, CA, U.S.A.) operated in negative ionization mode at 4 GHz high-resolution in a range from 50–1,000 m/z using published settings. All samples were analyzed in a single batch to minimize inter-batch variability, and always two technical replicates per sample were analyzed. The mobile phase was 60:40 isopropanol:water (v/v) and 1 mM NH_4_F at pH 9.0 supplemented with 10 nM hexakis(1H-, 1H-, 3H-tetrafluoropropoxy)phosphazine and 80 nM taurocholic acid for online mass correction. Spectral processing and ion annotation based on accurate mass within 0.005 Da using for each species a list of metabolites derived from reactants of genome-encoded enzymes based on the KEGG database [35] and accounting for [M-H]^−^ and [M-H, 1x^12^C->^13^C]^−^ species was performed using Matlab R2015a (The Mathworks, Nattick, MA, U.S.A.) as described previously [33]. Metabolomics data was quantile-normalized to account for variations in extracted biomass, and either log_2_ fold-changes of normalized ion intensities relative to unstressed conditions or *Z*-scores for each ion across all species (the abundance of an ion in a species minus the mean abundance of this ion divided by its standard deviation) were calculated as indicated to determine relative quantities.

### Statistical and computational analysis

Statistical and computational analysis of data was performed using Matlab R2015a (The Mathworks) and functions embedded in the Bioinformatics and Statistics toolboxes. The types of statistical tests used and the returned *P*-values are indicated when referring to these tests. Multiple testing correction was applied when indicated using Storey’s and Tibshirani’s method of false discovery rate estimation [60].

## Supporting Information

S1 Data Global metabolic responses to salt stress in fifteen species. (XLSX)

S2 Data Correlation of metabolite response magnitudes with species’ salt tolerance. (XLSX)

S1 Fig Analysis of growth inhibition by salt. Exponential growth rates of microbes under sustained salt stress were determined by absorbance (bacteria) or turbidity (yeasts) measurements in complex media at 30°C and normalized to respective growth rates in unstressed conditions. Human cell lines were cultivated at 37°C for 72 h at indicated salt concentrations and cell count was determined based on Hoechst fluorescence measurements and normalization to respective cell counts in unstressed conditions. (A) *A*. *tumefaciens*. (B) *B*. *subtilis*. (C) *C*. *glutamicum*. (D) *E*. *coli*. (E) *L*. *casei*. (F) *M*. *smegmatis*. (G) *P*. *versutus*. (H) *P*. *fluorescens*. (I) *P*. *putida*. (J) *R*. *sphaeroides*. (K) *S*. *cerevisiae*. (L) *S*. *pombe*. (M) *S*. *meliloti*. (N) *Z*. *mobilis*. (O) *H*. *sapiens* HDF. (P) *H*. *sapiens* MCF7. (Q) Legend and axis labels for panels A-P. n = 2 for microbes and n = 4 for human cell lines.
(TIF)

S2 Fig Intracellular pools of known osmoprotectants. (A) *E*. *coli* grown in minimal glucose medium, intracellular data from [43]. (B) *S*. *cerevisiae* grown in minimal glucose medium, dry-weight specific data from [42] converted to intracellular concentrations assuming a cytoplasmic volume of 40 fL and a cellular dry weight of 30 pg/cell. Only compounds quantified in both studies are considered in panels A and B to account for differential analytical coverage. These compounds are listed below the legend. (C) Total intracellular concentrations of the quantified osmoprotectants in both studies. Compounds were classified as known osmoprotectants according to the DEOP database [36].
(TIF)

S3 Fig Natural habitat, cell wall structure and salt tolerance have no dominant influence on the global metabolic salt stress response. Principal component analysis (PCA) was performed based on log_2_ metabolite ion fold-changes upon low (IC_10_, L), medium (IC_25_, M) or high (IC_50_, H) salt stress relative to unstressed controls. For each species the three stress intensity points are connected by triangular patches for visualization purposes. Patches and labels are colored according to the respective legends in the panels as defined in Fig 1A. Classification of species based on (A) natural habitat; (B) cell wall thickness and (C) salt tolerance (IC_50_ < 500 mM NaCl = low; 500 ≤ IC_50_ ≤ 1,000 mM = medium; IC_50_ > 1,000 mM = high). The underlying loading plot with highlighted selected metabolites is shown in Fig 3B.
(TIF)

S4 Fig Quantification of cell numbers by Hoechst-staining of nuclei. Calibration curves were generated by diluting known cell numbers in PBS, applying Hoechst staining and measuring fluorescence intensity at wavelengths of 350 nm (excitation) and 460 nm (emission). Data is shown as mean and standard deviation of six replicates. A linear fit was applied to points in the unsaturated signal range. (A) *H*. *sapiens* HDF cells. (B) *H*. *sapiens* MCF7 cells.
(TIF)

S1 Table Characteristics of investigated species. (PDF)

S2 Table Salt tolerances of investigated species. (PDF)
