# Supplementary figures and images for: Global Metabolic Responses to Salt Stress in Fifteen Species
Source: PLoS One. 2016 Feb 5;11(2):e0148888. doi: 10.1371/journal.pone.0148888 (PMC4743995; doi:10.1371/journal.pone.0148888)

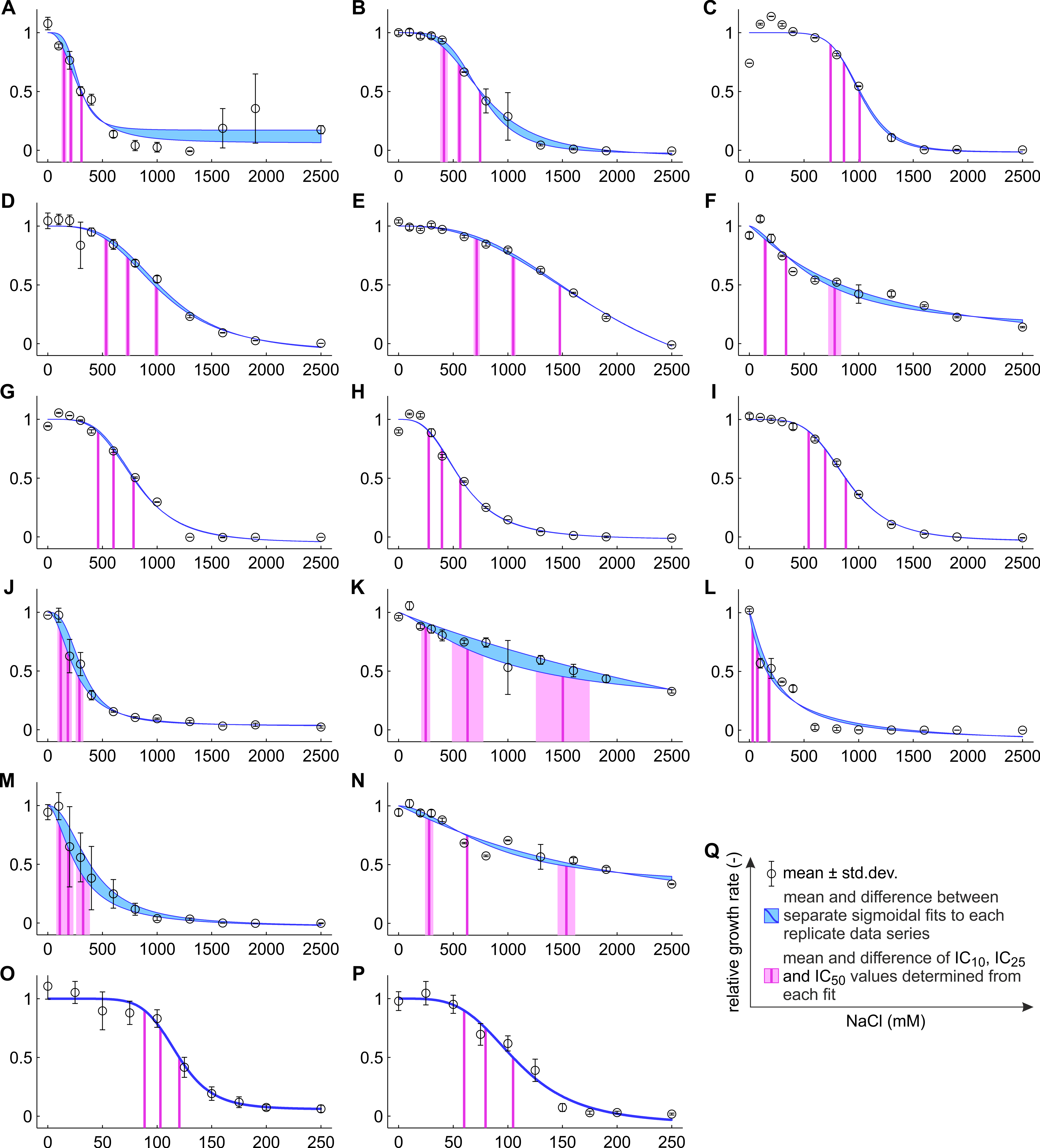

Supplement: S1 Fig — Exponential growth rates of microbes under sustained salt stress were determined by absorbance (bacteria) or turbidity (yeasts) measurements in complex media at 30°C and normalized to respective growth rates in unstressed conditions. Human cell lines were cultivated at 37°C for 72 h at indicated salt concentrations and cell count was determined based on Hoechst fluorescence measurements and normalization to respective cell counts in unstressed conditions. (A) A. tumefaciens. (B) B. subtilis. (C) C. glutamicum. (D) E. coli. (E) L. casei. (F) M. smegmatis. (G) P. versutus. (H) P. fluorescens. (I) P. putida. (J) R. sphaeroides. (K) S. cerevisiae. (L) S. pombe. (M) S. meliloti. (N) Z. mobilis. (O) H. sapiens HDF. (P) H. sapiens MCF7. (Q) Legend and axis labels for panels A-P. n = 2 for microbes and n = 4 for human cell lines. (TIF) [file pone.0148888.s003.tif]

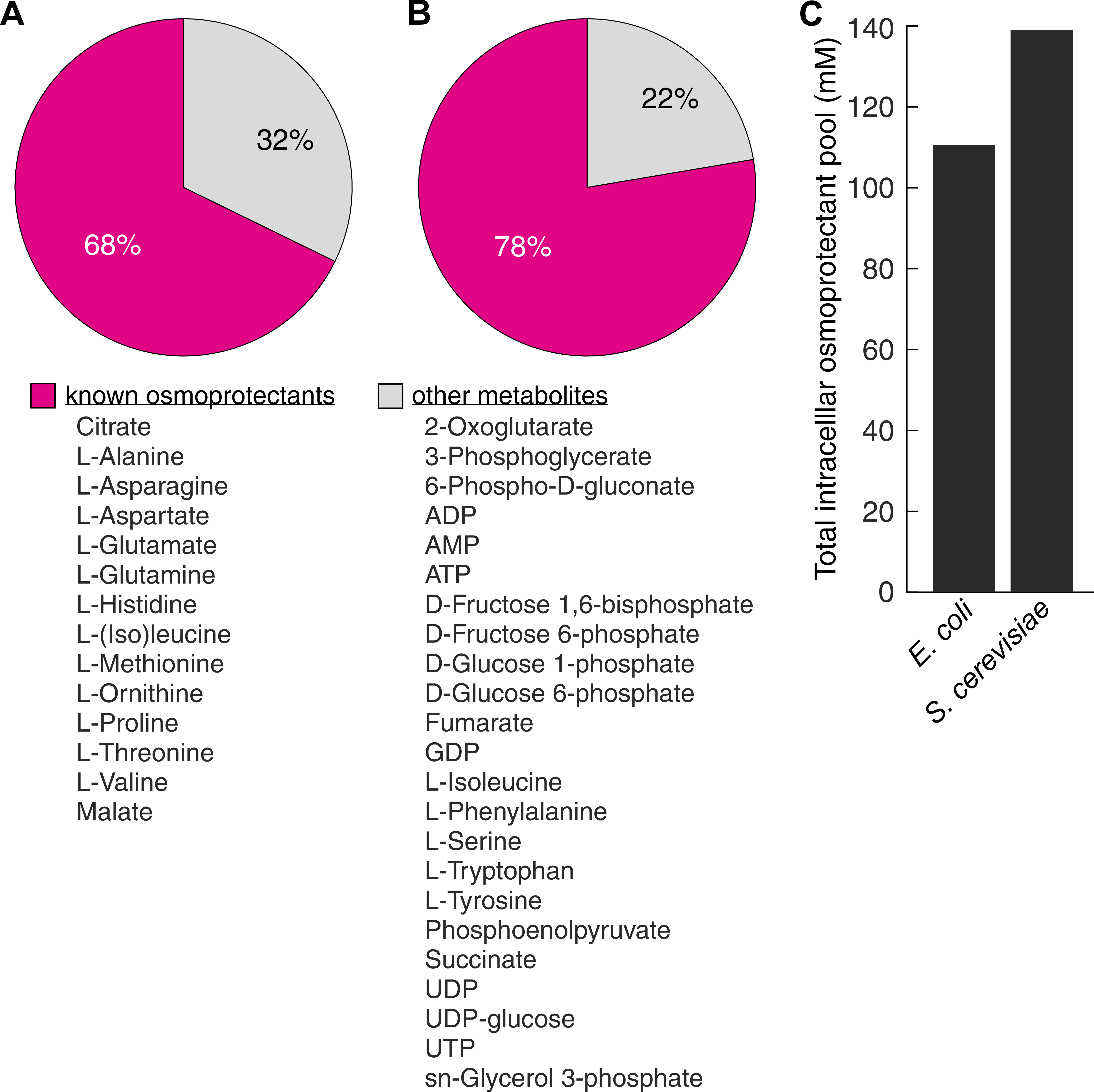

Supplement: S2 Fig — (A) E. coli grown in minimal glucose medium, intracellular data from [43]. (B) S. cerevisiae grown in minimal glucose medium, dry-weight specific data from [42] converted to intracellular concentrations assuming a cytoplasmic volume of 40 fL and a cellular dry weight of 30 pg/cell. Only compounds quantified in both studies are considered in panels A and B to account for differential analytical coverage. These compounds are listed below the legend. (C) Total intracellular concentrations of the quantified osmoprotectants in both studies. Compounds were classified as known osmoprotectants according to the DEOP database [36]. (TIF) [file pone.0148888.s004.tif]

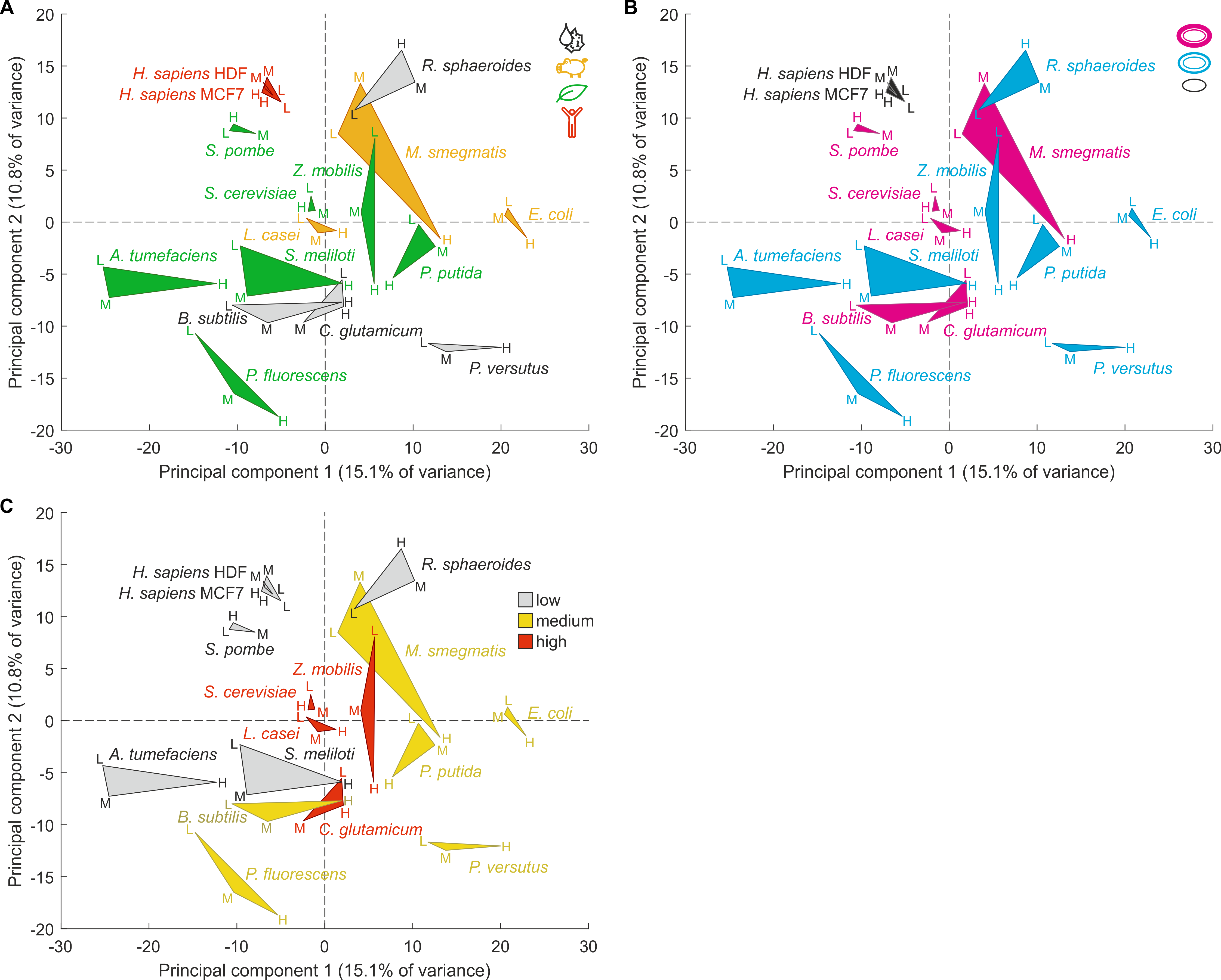

Supplement: S3 Fig — Principal component analysis (PCA) was performed based on log2 metabolite ion fold-changes upon low (IC10, L), medium (IC25, M) or high (IC50, H) salt stress relative to unstressed controls. For each species the three stress intensity points are connected by triangular patches for visualization purposes. Patches and labels are colored according to the respective legends in the panels as defined in Fig 1A. Classification of species based on (A) natural habitat; (B) cell wall thickness and (C) salt tolerance (IC50 < 500 mM NaCl = low; 500 ≤ IC50 ≤ 1,000 mM = medium; IC50 > 1,000 mM = high). The underlying loading plot with highlighted selected metabolites is shown in Fig 3B. (TIF) [file pone.0148888.s005.tif]

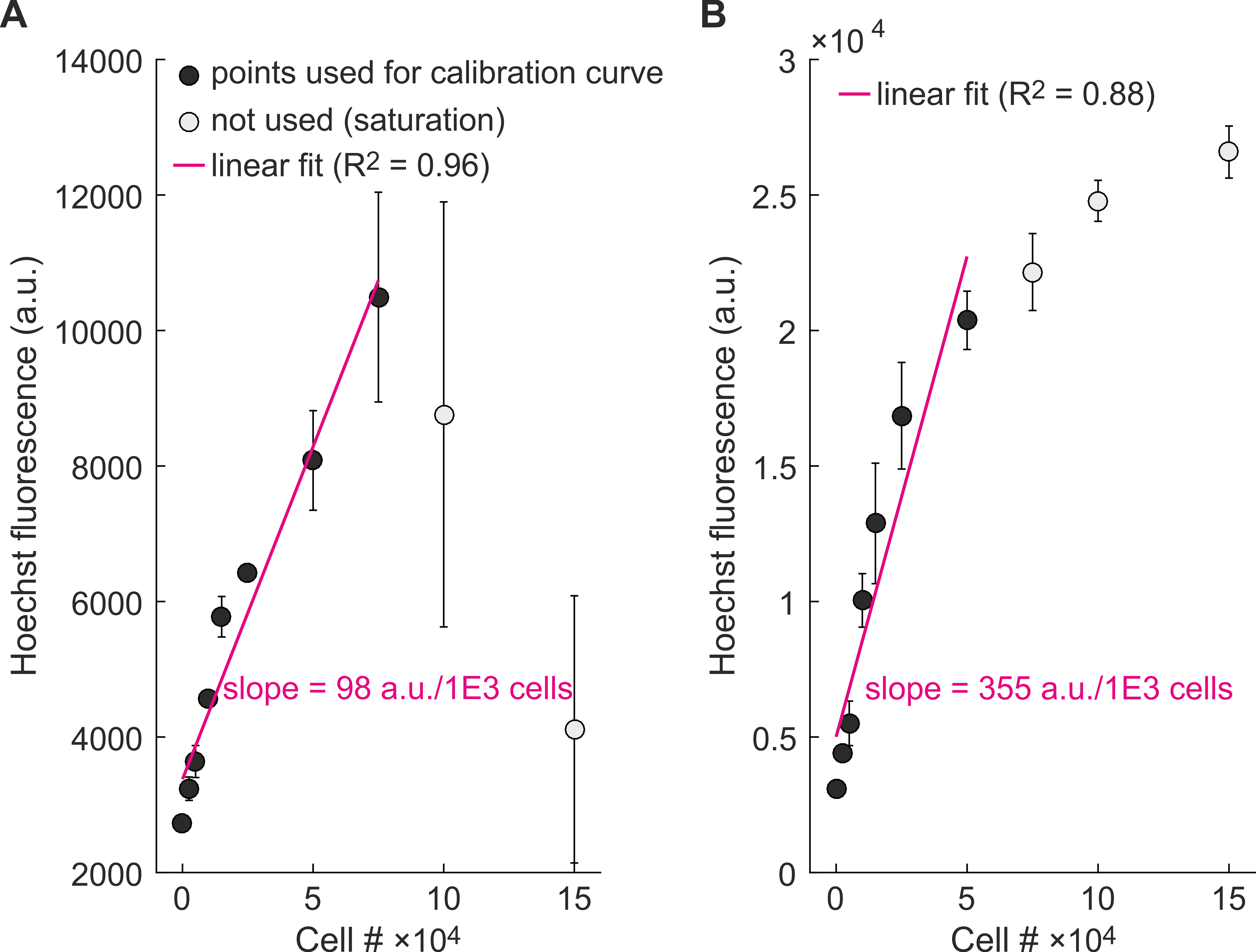

Supplement: S4 Fig — Calibration curves were generated by diluting known cell numbers in PBS, applying Hoechst staining and measuring fluorescence intensity at wavelengths of 350 nm (excitation) and 460 nm (emission). Data is shown as mean and standard deviation of six replicates. A linear fit was applied to points in the unsaturated signal range. (A) H. sapiens HDF cells. (B) H. sapiens MCF7 cells. (TIF) [file pone.0148888.s006.tif]
