# Supplementary material for: Global Metabolic Responses to Salt Stress in Fifteen Species
Source: PLoS One. 2016 Feb 5;11(2):e0148888. doi: 10.1371/journal.pone.0148888 (PMC4743995; doi:10.1371/journal.pone.0148888)
Supplement: S1 Table — (PDF) [file pone.0148888.s007.pdf]

S1 Table. Characteristics of investigated species.

genus, species, strain/cell line	cell envelope characteristics	habitat or tissue	main known compatible solutes (*de novo* synthesis induced upon osmotic stress; excluding compounds only taken up from environment)	main isoprenoid quinone[a]	source or reference
Homo sapiens HDF	No cell wall	Primary neonatal dermal fibroblast cells	sorbitol, inositol, betaine, taurine, glycerophosphocholine [1]	Q10 [2]	Cell Applications Inc., catalog # 106-05n
Homo sapiens MCF7	No cell wall	Epithelial breast cancer cell line	sorbitol, inositol, betaine, taurine, glycerophosphocholine [1]	Q10 [2]	DSMZ # ACC 115
Saccharomyces cerevisiae strain Sa-07140	Glucan, mannan, chitin in cell wall	Surface of plants and ripening fruit	glycerol [3], trehalose [4]	Q6 [5]	DSMZ # 70449
Schizosaccharomyces pombe strain 975	Glucan, galactomannan, chitin in cell wall	Surface of plants and ripening fruit	glycerol [6]	Q10 [5]	DSMZ # 70577
Mycobacterium smegmatis strain GA 735	Gram-positive, mycolic acids (acid-fast)	Aquatic environments, human skin	ectoine, hydroxyectoine [7]	K9 [8]	DSMZ # 43756
Corynebacterium glutamicum strain 534	Gram-positive, mycolic acids	Soil, water, surface of plants	proline [9], trehalose [10]	K9 [8]	DSMZ # 20300
Bacillus subtilis strain 168	Gram-positive	Soil	proline [11]	K7 [8]	DSMZ # 23778
Lactobacillus casei strain 03, 7	Gram-positive	Milk, water	none [12]	K[b] [8]	DSMZ # 20011
Agrobacterium tumefaciens strain EX 3.24.2 (L 624)	Gram-negative	Soil, plant surface	mannosucrose [13]	Q10 [8]	DSMZ # 30147
Sinorhizobium meliloti strain 3D0a2	Gram-negative	Soil, plant roots, plant nodules	glutamate [14], N-acetyl-glutaminylglutamate [15]	Q10[c] [16]	DSMZ # 30135
Paracoccus versutus strain A2	Gram-negative	Mud, soil	*no relevant publications found*	Q10 [17]	DSMZ # 582
Rhodobacter sphaeroides strain ATH 2.4.1	Gram-negative	Deep lakes, stagnate waters	trehalose [18]	Q10 [8]	DSMZ # 158
Zymomonas mobilis strain IMG 1655	Gram-negative	Plant saps, soil	sorbitol [19]	Q10 [20]	DSMZ # 424
Escherichia coli strain BW25113	Gram-negative	Gastrointestinal tract of mammals	trehalose [21], glutamate [22]	Q8 [8]	[23]
Pseudomonas fluorescens biovar I (742)	Gram-negative	Soil, water, surface of plants	glutamate, N-acetyl-glutaminylglutamine amide, trehalose [15]	Q9 [8]	DSMZ # 50090
Pseudomonas putida strain 90	Gram-negative	Soil	N-acetyl-glutaminylglutamine amide, mannitol [24]	Q9 [8]	DSMZ # 291

[a]Q, ubiquinone; K, menaquinone. Numbers indicate subunit count of isoprenyl chain

[b]Length of isoprenyl chain unknown

[c]Inferred from quinone composition of two related species of the genus *Sinorhizobium*

S1 Table References

1. Burg MB. Molecular basis of osmotic regulation. Am J Physiol. 1995;268: F983–F996.
2. Nowicka B, Kruk J. Occurrence, biosynthesis and function of isoprenoid quinones. Biochim Biophys Acta - Bioenerg. Elsevier B.V.; 2010;1797: 1587–1605. doi:10.1016/j.bbabio.2010.06.007
3. Albertyn J, Hohmann S, Thevelein JM, Prior B a. GPD1, which encodes glycerol-3-phosphate dehydrogenase, is essential for growth under osmotic stress in Saccharomyces cerevisiae, and its expression is regulated by the high-osmolarity glycerol response pathway. Mol Cell Biol. 1994;14: 4135–44. doi:10.1128/MCB.14.6.4135
4. Hounsa C-G, Brandt E V., Thevelein J, Hohmann S, Prior BA. Role of trehalose in survival of Saccharomyces cerevisiae under osmotic stress. Microbiology. 1998;144: 671–680. doi:10.1099/00221287-144-3-671
5. Kawamukai M. Biosynthesis, Bioproduction and Novel Roles of Ubiquinone. J Biosci Bioeng. 2002;94: 511–517. Available: http://www.sciencedirect.com/science/article/pii/S1389172302801888
6. Aiba H, Yamada H, Ohmiya R, Mizuno T. The osmo-inducible gpd1+ gene is a target of the signaling pathway involving Wis1 MAP-kinase kinase in fission yeast. FEBS Lett. 1995;376: 199–201. doi:10.1016/0014-5793(95)01277-4
7. Ofer N, Wishkautzan M, Meijler M, Wang Y, Speer A, Niederweis M, et al. Ectoine Biosynthesis in Mycobacterium smegmatis. Appl Environ Microbiol. 2012;78: 7483–7486. doi:10.1128/AEM.01318-12
8. Collins MD, Jones D. Distribution of Isoprenoid Quinone Structural Types in Bacteria and Their Taxonomic Implications. Microbiol Mol Biol Rev. 1981;42: 316–354.
9. Guillouet S, Engasser JM. Growth of Corynebacterium glutamicum in glucose-limited continuous cultures under high osmotic pressure. Influence of growth rate on the intracellular accumulation of proline, glutamate and trehalose. Appl Microbiol Biotechnol. 1995;44: 496–500. doi:10.1007/BF00169950
10. Wolf A, Krämer R, Morbach S. Three pathways for trehalose metabolism in Corynebacterium glutamicum ATCC13032 and their significance in response to osmotic stress. Mol Microbiol. 2003;49: 1119–1134. doi:10.1046/j.1365-2958.2003.03625.x
11. Brill J, Hoffmann T, Bleisteiner M, Bremer E. Osmotically Controlled Synthesis of the Compatible Solute Proline Is Critical for Cellular Defense of Bacillus subtilis against High Osmolarity. J Bacteriol. 2011;193: 5335–5346. doi:10.1128/JB.05490-11
12. Poolman B, Glaasker E. Regulation of compatible solute accumulation in bacteria. Mol Microbiol. 1998;29: 397–407. doi:10.1046/j.1365-2958.1998.00875.x
13. Fernandez-Aunión C, Hamouda T Ben, Iglesias-Guerra F, Argandoña M, Reina-Bueno M, Nieto JJ, et al. Biosynthesis of compatible solutes in rhizobial strains isolated from Phaseolus vulgaris nodules in Tunisian fields. BMC Microbiol. 2010;10: 192. doi:10.1186/1471-2180-10-192
14. Botsford JL, Lewis T a. Osmoregulation in Rhizobium meliloti: Production of glutamic acid in response to osmotic stress. Appl Environ Microbiol. 1990;56: 488–494.
15. Smith LT, Smith GM. An osmoregulated dipeptide in stressed Rhizobium meliloti. J Bacteriol. 1989;171: 4714–7. Available: http://www.pubmedcentral.nih.gov/articlerender.fcgi?artid=210271&tool=pmcentrez&rendertype=abstract
16. Ogasawara M, Suzuki T, Mutoh I, Annapurna K, Arora NK, Nishimura Y, et al. Sinorhizobium indiaense sp. nov. and Sinorhizobium abri sp. nov. Isolated from Tropical Legumes, Sesbania rostrate and Abrus precatorius, Respectively. Symbiosis. 2003;34: 53–68. doi:10.1017/CBO9781107415324.004
17. Lipski A, Klatte S, Bendinger B, Altendorf K. Differentiation of gram-negative, nonfermentative bacteria isolated from biofilters on the basis of fatty acid composition, quinone system, and physiological reaction profiles. Appl Environ Microbiol. 1992;58: 2053–2065.
18. Tsuzuki M, Moskvin O V., Kuribayashi M, Sato K, Retamal S, Abo M, et al. Salt Stress-Induced Changes in the Transcriptome, Compatible Solutes, and Membrane Lipids in the Facultatively Phototrophic Bacterium Rhodobacter sphaeroides. Appl Environ Microbiol. 2011;77: 7551–7559. doi:10.1128/AEM.05463-11
19. Loos H, Krämer R, Sahm H, Sprenger G a. Sorbitol promotes growth of Zymomonas mobilis in environments with high concentrations of sugar: evidence for a physiological function of glucose-fructose oxidoreductase in osmoprotection. J Bacteriol. 1994;176: 7688–7693.
20. Strohdeicher M, Neuss B, Bringer-Meyer S, Sahm H. Electron transport chain of Zymomonas mobilis. Arch Microbiol. 1990;154: 536–543.
21. Giaever HM, Styrvold OB, Kaasen I, Strom AR. Biochemical and genetic characterization of Osmoregulatory Trehalose Synthesis in Escherichia coli. J Bacteriol. 1988;170: 2841–2849.
22. Record MT, Courtenay ES, Cayley DS, Guttman HJ. Responses of E. coli to osmotic stress: Large changes in amounts of cytoplasmic solutes and water. Trends Biochem Sci. 1998;23: 143–148. doi:10.1016/S0968-0004(98)01196-7
23. Yamamoto N, Nakahigashi K, Nakamichi T, Yoshino M, Takai Y, Touda Y, et al. Update on the Keio collection of Escherichia coli single-gene deletion mutants. Mol Syst Biol. 2009;5: 335. doi:10.1038/msb.2009.92
24. Kets EP, Galinski E a, de Wit M, de Bont J a, Heipieper HJ. Mannitol, a novel bacterial compatible solute in Pseudomonas putida S12. J Bacteriol. 1996;178: 6665–70. Available: http://www.pubmedcentral.nih.gov/articlerender.fcgi?artid=178559&tool=pmcentrez&rendertype=abstract
