# Supplementary material for: Global Metabolic Responses to Salt Stress in Fifteen Species
Source: PLoS One. 2016 Feb 5;11(2):e0148888. doi: 10.1371/journal.pone.0148888 (PMC4743995; doi:10.1371/journal.pone.0148888)
Supplement: S2 Table — (PDF) [file pone.0148888.s008.pdf]

S2 Table. Salt tolerance of investigated species.

**species/cell line**	**IC_{10}** mean ± std.dev. *(mM NaCl)*	**IC_{25}** mean ± std.dev. *(mM NaCl)*	**IC_{50}** mean ± std.dev. *(mM NaCl)*
Homo sapiens HDF	89 ± 0	103 ± 0	120 ± 0
Homo sapiens MCF7	60 ± 0	80 ± 0	105 ± 0
Escherichia coli	534 ± 30	732 ± 30	995 ± 30
Pseudomonas putida	543 ± 4	693 ± 4	883 ± 4
Pseudomonas fluorescens	277 ± 5	397 ± 5	566 ± 4
Rhodobacter sphaeroides	119 ± 44	184 ± 51	290 ± 50
Paracoccus versutus	460 ± 14	602 ± 12	785 ± 9
Sinorhizobium meliloti	110 ± 46	189 ± 65	323 ± 88
Agrobacterium tumefaciens	149 ± 34	211 ± 27	308 ± 7
Zymomonas mobilis	280 ± 53	628 ± 14	1535 ± 115
Saccharomyces cerevisiae	249 ± 58	632 ± 203	1503 ± 349
Schizosaccharomyces pombe	30 ± 14	74 ± 23	180 ± 25
Lactobacillus casei	714 ± 41	1050 ± 30	1475 ± 12
Bacillus subtilis	415 ± 50	557 ± 30	746 ± 7
Corynebacterium glutamicum	743 ± 0	866 ± 4	1010 ± 9
Mycobacterium smegmatis	145 ± 25	335 ± 0	781 ± 85
